# *De novo* transcriptome analysis of *Perna viridis* highlights tissue-specific patterns for environmental studies

**DOI:** 10.1186/1471-2164-15-804

**Published:** 2014-09-19

**Authors:** Priscilla TY Leung, Jack CH Ip, Sarah ST Mak, Jian Wen Qiu, Paul KS Lam, Chris KC Wong, Leo L Chan, Kenneth MY Leung

**Affiliations:** The Swire Institute of Marine Science and School of Biological Sciences, The University of Hong Kong, Pokfulam, Hong Kong China; State Key Laboratory in Marine Pollution, City University of Hong Kong, Tat Chee Avenue, Kowloon, Hong Kong, China; Department of Biology, Hong Kong Baptist University, Kowloon, Hong Kong, China; Department of Biology and Chemistry, City University of Hong Kong, Tat Chee Avenue, Kowloon, Hong Kong, China

**Keywords:** *De novo* transcriptome, *Perna viridis*, Mussel, RNA-seq, Tissue specificity, Biomonitor

## Abstract

**Background:**

The tropical green-lipped mussel *Perna viridis* is a common biomonitor throughout the Indo-Pacific region that is used for environmental monitoring and ecotoxicological investigations. However, there is limited molecular data available regarding this species. We sought to establish a global transcriptome database from the tissues of adductor muscle, gills and the hepatopancreas of *P. viridis* in an effort to advance our understanding of the molecular aspects involved during specific toxicity responses in this sentinel species.

**Results:**

Illumina sequencing results yielded 544,272,542 high-quality filtered reads. After *de novo* assembly using Trinity, 233,257 contigs were generated with an average length of 1,264 bp and an N50 length of 2,868 bp; 192,879 assembled transcripts and 150,111 assembled unigenes were obtained after clustering. A total of 93,668 assembled transcripts (66,692 assembled genes) with putative functions for protein domains were predicted based on InterProScan analysis. Based on similarity searches, 44,713 assembled transcripts and 25,319 assembled unigenes were annotated with at least one BLAST hit. A total of 21,262 assembled transcripts (11,947 assembled genes) were annotated with at least one well-defined Gene Ontology (GO) and 5,131 assembled transcripts (3,181 assembled unigenes) were assigned to 329 Kyoto Encyclopedia of Genes and Genomes (KEGG) pathways. The quantity of assembled unigenes and transcripts obtained from male and female mussels were similar but varied among the three studied tissues, with the highest numbers recorded in the gills, followed by the hepatopancreas, and then the adductor muscle. Multivariate analyses revealed strong tissue-specific patterns among the three different tissues, but not between sexes in terms of expression profiles for annotated genes in various GO terms, and genes associated with stress responses and degradation of xenobiotics. The expression profiles of certain selected genes in each tissue type were further validated using real-time quantitative polymerase chain reaction assays and a similar tissue-specific trend was seen.

**Conclusions:**

The extensive sequence data generated from this study will provide a valuable molecular resource for facilitating environmental studies with *P. viridis*, and highlight the importance of tissue-specific approaches in the future.

**Electronic supplementary material:**

The online version of this article (doi:10.1186/1471-2164-15-804) contains supplementary material, which is available to authorized users.

## Background

Over the past 30 years, mussels have served as useful biomonitors in assessing the quality of our marine ecosystems with respect to the impact of chemicals [1]. Mytilid mussels, in particular *Mytilus* species, have been extensively applied to environmental monitoring programs and toxicological studies in temperate regions [2]. More recently, a tropical/subtropical member of mytilids, the green-lipped mussel *Perna viridis*, has been adopted as a biomonitor throughout the Indo-Pacific region [3] for assessing a wide range of chemical pollutants [4–8]. Because of its wide distribution, and high tolerance to environmental stresses, this mussel is also regarded as highly invasive and a biofouling species [9].

In conventional biomonitoring, the body burden of common pollutants and a limited number of physiological and molecular biomarkers are used as the end-points for assessing the effects of chemical pollution on mussels [3]. To understand the toxic effects of chemical pollutants, and to develop an early warning signal of pollutant stress on a biomonitor, it is essential to have an in-depth knowledge of their toxic mechanisms at a molecular level. However, like many other non-model marine organisms, the limited genomic data available for *P. viridis* has hindered studies attempting to elucidate molecular mechanisms related to specific toxic stress responses.

A transcriptome of a given organism refers to a complete set of transcripts present in a cell type at a specific developmental stage or physiological condition [10]. The underlying molecular mechanisms and phenotypic plasticity of an organism against environmental influences can be interpreted from the dynamics of its transcriptome. Given the advent of next-generation sequencing (NGS) technology, high throughput RNA-sequencing analysis of transcripts with greater gene coverage, higher sensitivity, and better reproducibility of transcriptomes that are highly complex, can now be applied to any genome [10]. RNA-sequencing analysis has been applied to several marine bivalve species, including the Mediterranean mussel *Mytilus galloprovincialis* [11], the deep-sea hydrothermal vent mussel *Bathymodiolus azoricus* [12], the Manila clam *Ruditapes philippinarum* [13], the Yesso scallop *Mizuhopecten yessoensis* (formerly known as *Patinopecten yessoensis*) [14], the pearl oyster *Pinctada fucata* [15] and the Pacific oyster *Crassostrea gigas* [16]. Some of these studies have highlighted the species specificity of the transcriptome, and also demonstrated specific transcript profiles among different tissues. The extensive and novel genomic data generated from these non-model animals have revealed much about their underlying molecular mechanisms in response to different environmental stressors [13, 17].

Compared with other mytilids, genomic and protein databases are largely unavailable for *P. viridis*. To date, the number of sequence entries available for this species in the National Center for Biotechnology Information (NCBI) is limited, with 451 nucleotide sequences, 4 expressed sequence tags (ESTs), 252 protein sequences, and 1 high-throughput DNA and RNA sequence read archive (SRA) (Table 1, Data search performed on 15 May 2014). Molecular characterization of stress-associated genes, such as metallothioneins [18] and glutathione S-transferases [19], and the adhesion associated proteins such as the 3, 4-dihydroxyphenyl-L-alanine (DOPA)-containing proteins from the byssus threads [20] have been conducted for *P. viridis*. A recent study revealed the adhesive byssal complex and various adhesive proteins in the foot tissue of *P. viridis*, using RNA-sequencing and proteomics [21]. A number of microsatellite markers were also characterized for genetic investigations [22, 23]. *P. viridis* has the shortest reported mitochondrial genome (16,014 bp) among marine mussels, and differs from that of other *Mytilus* species in terms of genome organization [24]. A cytogenetic study suggested that *P. viridis* could be a more primitive species of the Mytilidae, that diverged from other members of the *Perna* and *Mytilus* genera [25]. Such distinctions indicate that the existing genomic data from other Mytilidae member may not be suitable to serve as a basis for homologous references in *P. viridis*.

**Table 1:** **Sequence entries for Mytilidae including** ***Perna viridis*** **and three** ***Mytilus*** **species in the NCBI database**

Mussel	EST entries	Nucleotide entries	Protein entries	SRA
Mytilidae	71,328	54,548	12,803	65
*Perna viridis*	4	451	252	1
*Mytilus californianus*	42,365	945	969	0
*Mytilus galloprovincialis*	19,756	41,167	2,417	16
*Mytilus edulis*	5,352	5,301	3,496	45

*Note*: Search was performed on 15 May 2014.

Given the importance of *P. viridis* to ecotoxicology and environmental applications, a comprehensive genomic database is urgently needed for this species to advance future marine environmental studies in the Indo-Pacific region. In the current study, we applied Illumina sequencing to characterize a *de novo* transcriptome assembled from a mixed sample of three target tissues (adductor muscle, gills and hepatopancreas) of male and female *P. viridis* collected from the field and from various designated exposure scenarios. Comparative analyses were also performed to determine any tissue- and sex-specific transcriptome profiles.

## Results and discussion

### Sequencing and assembly

An overall profile of the transcriptome of *P. viridis* was obtained through Illumina sequencing of six cDNA libraries (2 sexes × 3 tissues) and pooled mRNA samples from the adductor muscle, gills and hepatopancreas of male and female. The number of reads generated for each target tissue ranged from 84,607,612–102,182,018 with a Q30 values (denoting the accuracy of a base call to be 99.9%) ranging from 83.78–89.19%. The highest GC content was seen in the adductor muscle (42%), followed by the hepatopancreas (40%), and the gills (39%) (Additional file 1).

A global *de novo* transcriptome of *P. viridis* was assembled from the pool of the reads for the six cDNA libraries using Trinity software. We obtained 544,272,542 high-quality reads after filtering and subjecting to *de novo* assembly, generating 233,257 contigs. After clustering with CD-Hit, the resulting assembled transcriptome comprised 192,879 assembled transcripts and 150,111 assembled unigenes (Table 2). The lengths of the assembled transcripts ranged from 201–43,014 bp, with an average length of 1,264 bp and an N50 length of 2,868 bp (Table 2). The read data for the six samples was deposited in the NCBI SRA database (BioProject Accession Number SRP043984).

**Table 2:** **Summary of the assembled transcriptome of** ***Perna viridis***

Item	Number
Total number of filtered reads	544,272,542
Total number of assembled contigs	233,257
Number of assembled transcripts after clustering	192,879
Number of assembled unigenes after clustering	150,011
N50 (bp)	2,868
Minimum contig length (bp)	201
Maximum contig length (bp)	43,014
Average contig length (bp)	1,264
Total length in contigs (bp)	295,064,579

### Sequence abundance

**Figure 1:**
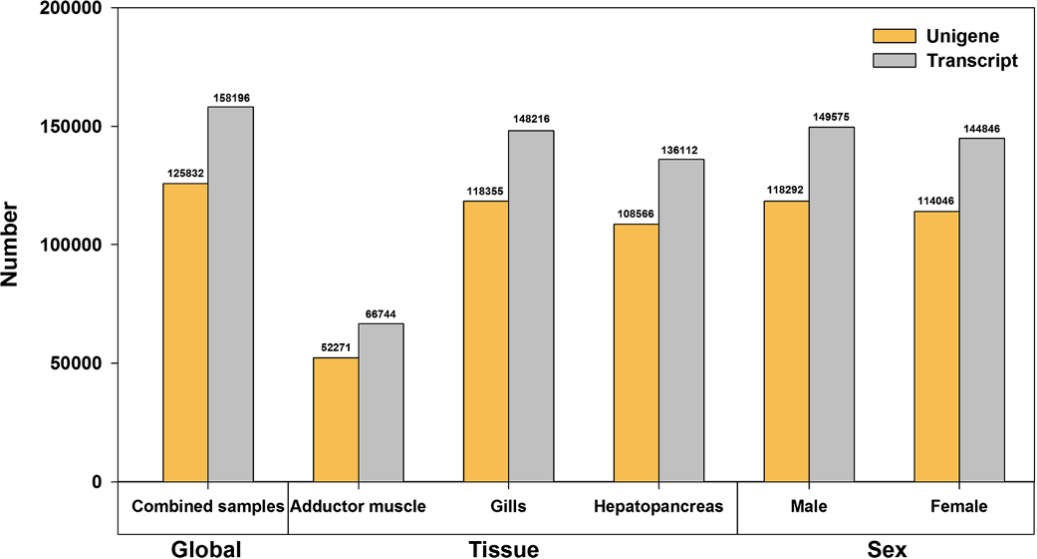
**Assembled unigenes and transcripts for combined samples, individual tissue types and by sex.** The expression level of assembled transcripts/unigenes was measured using fragments per kilobases per million reads (FPKM). An assembled transcript/unigene was considered expressed using an FPKM cut-off value ≥ 0.05.

The expression levels of assembled unigenes/transcripts were measured using fragments per kilobases per million reads (FPKM). The numbers of assembled unigenes/transcripts that were expressed (cut-off value at FPKM ≥ 0.05) in male and female *P. viridis* were similar, however strong variations were observed for unigene/transcript abundance among tissues (Figure 1). The largest transcriptome size was obtained from the gills, followed by the hepatopancreas, and then the adductor muscle. The numbers of assembled unigenes/transcripts in the gills and hepatopancreas were very similar, while those detected in the adductor muscle were comparatively low. A recent study outlined the potential errors in using whole organism or composite structures (organs) in RNA-sequencing analysis as a common practice for small invertebrates like insects or larvae. The same researchers demonstrated the significance of using a structure/organ of interest to correctly interpret specific expression of genes [26]. We have demonstrated the possibility of high variability in transcriptome sizes occurring in different tissues of *P. viridis*.

### Functional annotation

Annotation results for the non-redundant assembled transcripts/unigenes are summarized in Table 3. We performed a BLAST search against the NCBI nr database and the three molluscan EST databases; 44,713 assembled transcripts and 25,319 assembled genes with at least one BLAST hit were returned, with 41,686 assembled transcripts and 23,392 assembled unigenes that had been annotated by the NBCI nr database. The BLAST search of the 27,651 assembled transcripts had their best match with sequences from the Pacific oyster, *Crassostrea gigas* (Additional file 2).

**Table 3:** **Summary of the annotation results**

Item	Number	
	Transcript	Unigene
With BLAST hit	44,713	25,319
With InterProScan result	93,668	66,692
With GO annotation	21,262	11,947
With KEGG annotation	5,131	3,181
KEGG pathways involved	329	329

Annotation results from the BLAST search for *de novo* transcriptomes of the non-model species were shown to be highly dependent on the availability of annotated sequence information for a reference genome, and the sizes of their contig sequences [27, 28]. The number of transcripts obtained for *P. viridis* from this study was comparatively higher than for other non-model bivalve species subjected to NGS technology. There were 38,942 reported sequences (14,638 contigs plus 24,304 singletons) [14] and 14,240 transcripts in the Yesso scallop *Mizuhopecten yessoensis* [29] *,* 22,023 transcripts in the deep-sea hydrothermal vent mussel *Bathymodiolus azoricus* [12], 18,196 contigs in the striped venus *Chamelea gallina* [27], 9,747 transcripts in the Manila clam *Ruditapes philippinarum* [13] and 1,023 contigs in the Mediterranean mussel *Mytilus galloprovincialis* [11]. At the gene and transcript levels, the annotation results for *P. viridis* that we obtained were comparable to transcriptomes obtained from multiple organs and at various developmental stages of the Pacific oyster *C. gigas* (45,771 transcripts [17] and 28,027 protein-coding predicted genes [16]). The improvement in the annotation of assembled transcripts of *P. viridis* could be explained by the higher sequence coverage obtained because of better sequence length (average contig length: 1,264 bp; N50: 2,868 bp), and the substantial annotated genomic data available from the recently sequenced genome of *C. gigas* [16].

A total of 93,668 assembled transcripts and 66,692 assembled unigenes were predicted to have putative functions in protein domains based on InterProScan analysis. Gene Ontology (GO) functional terms were assigned according to NCBI nr annotations and the InterProScan results. From the global transcriptome, 21,262 assembled transcripts and 11,947 assembled unigenes were associated with at least one well-defined GO term. These were further classified into 31 functional groups (GO term of level 2) according to three main functional categories: biological process; cellular component; and molecular function (Additional file 3). The number of genes assigned to “metabolic process” (24.4%) was the highest under the biological process classification, while genes annotated with functions related to “cell” (64.8%) and “binding” (57.7%) were dominant for the cellular component and molecular function categories, respectively.

**Figure 2:**
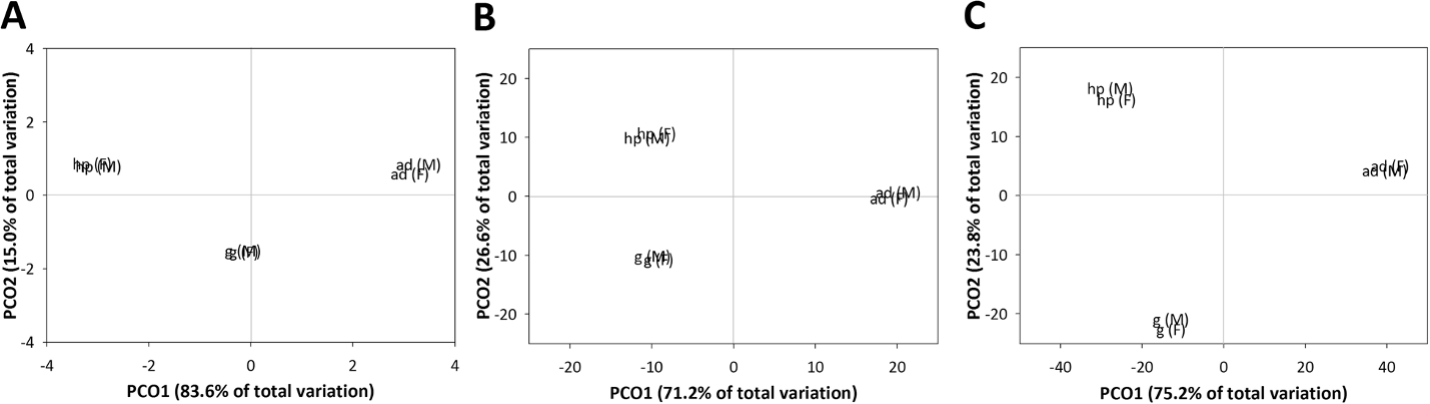
**Principle coordinate analysis plots for expression patterns of individual tissue types and by sex.** Variations in the expression profiles of **(A)** all genes annotated in the 31 various functional categories for level 2 gene ontology (GO) terms, **(B)** a subgroup of 187 genes involved in “response to stress” from GO functional categories, and **(C)** a subgroup of 96 genes involved in “xenobiotics biodegradation and metabolism” from KEGG pathways. Studied tissues were the adductor muscle (ad), gills (g) and hepatopancreas (hp). Male and female mussels are denoted as (M) and (F), respectively.

The composition of various GO terms for genes from individual tissue types and sexes can be seen in Additional file 4. Variations in the GO term compositions across the three tissue types and between the sexes were not apparent. Kinoshita et al. [15] also reported that there was no difference in GO composition of the annotated genes from the pallium, mental edge and pearl sac of the pearl oyster *Pinctada fucata*. However, a strong tissue-specific expression pattern was found among the three different tissues of *P. viridis* that we examined, without any apparent sex-specific patterns when multivariate analyses were applied (Figure 2A). A recent transcriptomic study on the Mediterranean mussel *M. galloprovincialis* also showed significant tissue-specific differences among four tissues with respect to transcript expression profiles in metabolic pathways [11]. We have successfully demonstrated that GO compositions did not vary among different tissues, but expression profiles exhibited distinctive tissue-specific patterns that were probably associated with the unique functions of the adductor muscle, gills, and the hepatopancreas.

For KEGG annotation, 5,131 assembled transcripts and 3,181 assembled unigenes were assigned to 329 KEGG pathways under the main groups: metabolism (1,126 genes); genetic information processing (928 genes); environmental information processing (597 genes); cellular processes (618 genes); organismal systems (746 genes); and human diseases pathway (874 genes) (Additional file 5). The functional annotations for GO terms and KEGG pathways offer a useful resource for future studies of specific pathways, cellular structures and protein functions of *P. viridis*. Subgroups of genes potentially involved in stress-associated responses and detoxification of xenobiotics are of particular interest to environmental researchers and ecotoxicologists.

### Functional genes related to stress-associated response and xenobiotic degradation

#### Tissue-specific expression patterns

**Figure 3:**
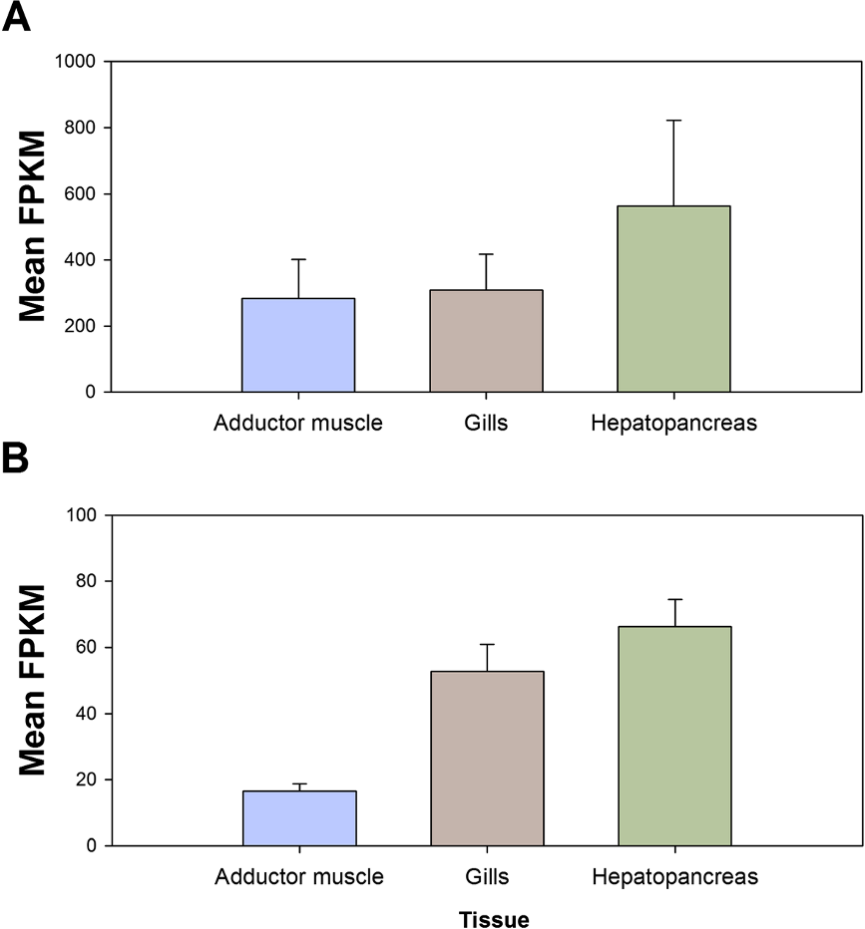
**Mean expression levels of the 20 most abundant annotated genes from individual tissue types.** Sub-groupings are based on the annotation to **(A)** “response to stress” and **(B)** “xenobiotics biodegradation and metabolism” from KEGG pathways. Expression levels are presented as mean FPKM values + S.E. for the top 20 most abundant annotated genes.

Based on GO term assignment and KEGG annotation, 187 assembled unigenes were annotated in “response to stress” under the level 3 classification for “biological process”, while 96 assembled unigenes were associated with the pathways of “xenobiotics biodegradation and metabolism” under the KEGG metabolism pathway. It is believed that genes from these two groups are highly relevant to the responses of *P. viridis* upon challenge with chemicals or other environmental factors. Multivariate analysis using principle coordinate analysis plot (PCO) revealed that expression patterns of the genes from these two categories exhibited distinct tissue-specific patterns (Figure 2B and C). Additionally, the top 20 most expressed assembled unigenes of each tissue type from the two categories representing > 90% and > 60% of genes expressing from the “response to stress” and “xenobiotics biodegradation and metabolism” groups, respectively, are summarized (Tables 4 and 5). Compared with the top 20 genes under “response to stress” in each tissue, relatively higher expression levels were reported for genes in the hepatopancreas. In “xenobiotics biodegradation and metabolism”, the top 20 genes in the gills and hepatopancreas exhibited higher expression levels than those in the adductor muscle (Figure 3).

**Table 4:** **20 most abundant annotated genes involved in “response to stress” from individual tissues**

BLAST top-hit description	FPKM*		
	Adductor muscle	Gills	Hepatopancreas
2'-5'-oligoadenylate synthetase 3	0.78	7.25	**38.09**
Activator of 90 kDa heat shock protein ATPase-like protein 1	**16.40**	**28.60**	**26.20**
B Chain B, Solution Structure Of S5a Uim-1 ubiquitin complex	**1693.91**	**1439.25**	**1004.83**
Catalase	10.49	18.55	**57.91**
Defensin-B	0.70	5.25	**5169.03**
Eosinophil peroxidase	0.28	**57.76**	1.04
Epididymal secretory glutathione peroxidase	0.01	1.76	**339.67**
Flap endonuclease 1-A	8.54	**20.45**	13.54
Glucose-regulated protein 94	**136.47**	**119.02**	0.19
Glutathione peroxidase 3	0.06	1.62	**872.96**
Growth arrest and DNA-damage-inducible protein GADD45 alpha	**29.06**	**54.65**	11.08
Growth arrest and DNA-damage-inducible protein GADD45 gamma	**32.61**	10.91	5.17
Heat shock factor binding protein 1	**23.82**	**354.84**	**138.25**
Heat shock protein 60	**15.56**	**57.83**	**51.70**
Heat shock protein 71	**1664.60**	**1676.22**	**1521.06**
Heat shock protein 90	**375.18**	**712.16**	**633.78**
Histone H3.3	**143.32**	**781.19**	**437.40**
Hypothetical protein CGI_10001640	**25.81**	**89.80**	**117.91**
Hypothetical protein CGI_10003818	0.22	0.10	**35.65**
Hypothetical protein CGI_10015993	**11.76**	1.71	5.98
Peroxidasin	0.22	**41.80**	0.27
Radical S-adenosyl methionine domain-containing protein 2	1.94	13.21	**26.31**
Ras-related C3 botulinum toxin substrate 1, partial	**15.05**	**26.70**	15.43
Retinal dehydrogenase	**21.62**	9.43	**43.25**
Selenium-dependent glutathione peroxidase	2.95	**26.33**	**68.00**
Small heat shock protein 22	**1023.03**	0.90	1.16
Small heat shock protein 24.1	**277.20**	**443.02**	**518.55**
Stress response protein nhaX	**61.17**	0.19	0.20
Unnamed protein product	5.23	**100.72**	13.63
UPS-like protein isoform 1	**26.24**	**42.42**	20.43
USP-like protein isoform 2	**23.19**	**73.67**	**120.01**
UV excision repair protein RAD23 homolog B-like	**48.10**	**46.30**	**36.59**

*Note*: *Expression levels are presented as FPKM values. The top 20 genes for each tissue are in boldface type.

**Table 5:** **20 most abundant annotated genes involved in “xenobiotics biodegradation and metabolism” in individual tissues**

BLAST top-hit description	FPKM*		
	Adductor muscle	Gills	Hepatopancreas
3-ketoacyl-CoA thiolase, mitochondrial	2.68	18.29	**44.68**
Acetyl- acetyltransferase mitochondrial	**16.01**	**21.24**	10.54
Alcohol dehydrogenase class-3	**53.46**	**30.34**	**81.41**
Aldehyde mitochondrial	**7.55**	**47.66**	**42.06**
ATPase family AAA domain-containing protein 2B	**11.38**	**39.79**	**56.63**
Carbonyl reductase	**16.1**	**68.81**	**59.44**
Cytochrome p450	0.62	11.38	**35.83**
Cytochrome p450 2c8	1.01	**40.79**	28.08
Cytochrome p450 2 k1-like	1.65	**27.14**	12.77
Cytochrome p450 3a-like isoform 1	2.76	4.11	**46.21**
Cytochrome p450 4f22	**14.48**	5.51	**35.58**
Dihydropyrimidinase	**21.62**	17.10	2.35
Dimethylaniline monooxygenase	1.52	4.14	**46.37**
Fumarylacetoacetase	**8.75**	18.67	8.63
Glutathione s-transferase alpha	**18.34**	**34.55**	**136.66**
Glutathione s-transferase pi 1	**16.12**	15.73	**44.15**
Glutathione s-transferase sigma 2	0.58	**176.58**	2.80
Glutathione s-transferase zeta	**20.35**	**21.10**	12.28
Glutathione-s-transferase omega class	0.54	**56.96**	**56.97**
Hydroxyacyl-coenzyme a mitochondrial	**16.01**	**20.28**	**56.62**
Hypothetical protein BRAFLDRAFT_86061	**19.69**	**65.68**	**70.07**
Hypothetical protein CAPTEDRAFT_149199	**8.35**	**68.49**	32.48
Microsomal glutathione s-transferase 2	5.76	**20.62**	26.62
Microsomal glutathione s-transferase 3	**16.04**	**44.71**	26.77
Microsomal glutathione-s-transferase	2.66	**38.78**	32.88
Monoamine oxidase A	**11.64**	2.73	12.58
Predicted protein	**9.98**	**99.6**	21.26
Regucalcin	0.07	0.26	**149.99**
Sodium- and chloride-dependent GABA transporter ine	0.62	**63.33**	**143.35**
Sorbitol dehydrogenase	**7.25**	3.22	**48.91**
Thymidine phosphorylase	4.77	13.95	**36.81**
Trans-1,2-dihydrobenzene-1,2-diol dehydrogenase	2.67	5.90	**38.77**
Trifunctional enzyme subunit beta, mitochondrial-like	**26.54**	**68.98**	**95.02**
Uridine-cytidine kinase-like 1	**9.03**	16.26	0.35

*Note*: *Expression levels are presented as FPKM values. The top 20 genes for each tissue are in boldface.

The expression of some of the selected assembled unigenes involved in stress-associated responses and xenobiotic degradation were further validated using quantitative PCR (qPCR) assays (Additional file 6). Most of these genes also demonstrated tissue-specific expression patterns with generally higher expression levels noticed in the hepatopancreas and/or the gills compared with those in the adductor muscle.

#### Heat-shock protein (HSP) genes

Heat-shock proteins (*HSPs*) are a group of stress-inducible chaperones that play crucial roles in modulating stress responses and offering cytoprotection [30]. *HSP*s in bivalves can be induced by various environmental stressors and chemical contaminants [31–34]. A number of *HSP*s were abundant among the top expressed genes in the “response to stress” category. These included *HSP71*, *HSP90*, *HSP60*, *HSP24.1*, *HSP22*, and other smaller HSPs. Tissue-specific expression patterns of *HSP*s have been reported in bivalve species. At basal levels, the *HSP70* gene in the oyster *Crassostrea hongkongensis* was more highly expressed in muscle than in other tissues such as gills, the digestive gland, mantle and heart. This gene was induced differentially and in a time-dependent manner in various tissues, with a faster and more potent response noticed in the digestive gland upon heat stress [32]. Expression of some of the identified *HSP* genes in individual tissues were also validated using qPCR analysis (Additional file 6). *HSP60* was expressed to a greater extent in the gills and hepatopancreas than in the adductor muscle, and was generally up-regulated by chemical exposure (Table 4, Additional file 6). The small HSP encoded by *HSP22* was present at high levels in the adductor muscle, and the protein encoded by *HSP24.1* was highly expressed in the gills and hepatopancreas but generally down-regulated by chemical stressors (Table 4, Additional file 6). A previous study suggested that some of the small *HSP*s in the mussel would play an essential chaperone role in maintaining cytoskeletal structural elements under adverse conditions induced by a combination of stressors when other *HSP*s, such as *HSP70* and *HSP90*, were suppressed [35]. Other genes encoding *HSP*s were also annotated in the present study (Additional file 7), including members from the *HSP40*, *HSP60*, *HSP70*, *HSP90* classes; a total of 19 members of *HSPs* were annotated for *P. viridis*.

#### Cytochrome P450 (CYP) genes

With respect to detoxification, members involved in the metabolism of xenobiotics *via* the cytochrome P450 pathway were particularly abundant among the top expressed genes under the KEGG pathways of “xenobiotics biodegradation and metabolism”, making up 40%, 60% and 50% of that category in the adductor muscle, gills and hepatopancreas of *P. viridis* respectively. The *CYP* genes encode one of the largest groups of enzymes that catalyze the oxidation of organic substances; they and play a central role in the Phase I detoxification system, and have a range of diverse functions in endogenous metabolism [36]. *CYP*s are classified into different clans according to homology between families; 11 clans were reported for animals [37], with at least four main clans (CYP2, CYP3, CYP4 clans and mitochondrial clan) being found in protostomes including mollusks [36, 38]. In our study, 14 families (33 members) of *CYP* genes were annotated for *P. viridis* (Additional file 8). Given the important role of *CYP* genes in the degradation of xenobiotics and other diverse functions, the understanding of this vast group of genes is important in environmental studies. In recent years, the increasing deposition of sequences through large-scale EST and genome projects has facilitated the identification of *CYP* genes in bivalve species. Zanette et al. [39] described *CYP* genes for some mussel and oyster species, included 58 members in *Mytilus californianus*, 12 members in *M. galloprovincialis*, and 14 members in *Crassostrea virginica*. Guo et al. [40] also identified 33 families of *CYP* genes, comprising 88 non-redundant members in the Zhikong scallop *Azumapecten farreri* (formerly known as *Chlamys farre*ri) *via* RNA sequencing analysis. With the completion of the *C. gigas* genome project, the number of *CYP* genes identified in oysters has further increased to 136 genes [16]. The majority of annotated *CYP* genes (11 members) in *P. viridis* were identified as members of clan 2 (Additional file 8).

The *CYP* genes of marine invertebrates, including mollusks, polychaetes and crustaceans, have been observed in a wide range of tissues and are generally enriched in organs associated with food processing [36, 41]. *CYP*s show tissue specificity with respect to their functions; for instance, *CYP*s in hepatic tissues are generally involved in the detoxification of a wide range of xenobiotics [36]. A member from *CYP* family 3 in the xenobiotics degradation pathway was one of the top 20 expressed genes in the hepatopancreas of *P. viridis*. The mRNA expression levels of this *CYP* were highest in this tissue (Additional file 6). A member of the *CYP12* family belonged to the mitochondrial clan and showed high expression levels in the gills, and was up-regulated by chemical stressors (Additional file 6). Although the function of *CYP12* genes has not been extensively studied in mussels, previous reports suggest that these genes are capable of metabolizing xenobiotics such as pesticides in arthropods [42, 43].

#### Glutathione S-transferase (GST) genes

Another predominant group contributing to the KEGG pathways of “xenobiotic biodegradation and metabolism” was the group of genes encoding GSTs. These genes comprised 25, 35 and 15% of the top expressed genes in the gills, adductor muscle, and hepatopancreas, respectively (Table 5). The GST proteins are a group of multifunctional enzymes that catalyze the conjunction and detoxification of xenobiotics during phase II enzyme detoxification [44]. Three major families of *GSTs* (cytosolic, mitochondrial, and microsomal) and at least 15 different classes of *GST*s (alpha, beta, delta, epsilon, kappa, lambda, mu, omega, phi, pi, sigma, tau, theta, zeta, and rho) have been identified in numerous organisms based on substrate specificity, and catalytic and immunological properties [44]. To date, only a limited number of *GST*s have been characterized in mussels. *GST* pi and omega were identified in *P. viridis* [19], while alpha, pi and sigma classes have been identified in *M. galloprovincialis* [45, 46], and pi class *GST* was found in *M. edulis* [47]. Characterization of *GSTs* has been conducted for other marine bivalves, such as oysters (*C. gigas* [48] and *C. ariakensis* [49]), and clams (*R. philippinarum* [50–52], *Laternula elliptica* [53, 54], *Mercenaria mercenaria* [55], *Solen grandis* [56] and *A. farreri* [57]). From our analysis 19 *GST*s were annotated for *P. viridis*, with most of them matching entries for *C. gigas* and *M. galloprovincialis* (Additional file 9). At least six classes of *GST*s (alpha, pi, sigma, omega, theta and zeta), along with microsomal *GST*s, and the C- and N-terminal domains were annotated for *P. viridis* (Additional file 9).

The tissue-specific expression patterns of *GST* genes in bivalves have been previously shown [19, 51]. A recent study on *P. viridis* showed that *GST* pi and omega classes showed higher transcriptional levels in the hepatopancreas than in the gonad, gills and mantle [19]. It was also reported that *GST* pi and *GST* sigma 1 in *M. galloprovincialis* were more highly expressed in the hepatopancreas than in the gills; *GSTs* alpha, sigma 2 and sigma 3 were found to be more abundant in hemocytes than in the hepatopancreas, gills, mantle, gonad and muscle [45, 46]. We also noticed strong tissue-specific expression patterns for the *GST* genes of *P. viridis* (Table 5). These strong tissue-specific expression patterns were also demonstrated by gene expression analysis for some of the selected *GST* members from the three tissues we examined (Additional file 6). Studies involving microarrays have also shown that differential expression of various *GST* genes from the gills and digestive gland of *R. philippinarum* were detected in samples collected from polluted areas [13]. Our novel findings regarding various *GST* genes in *P. viridis* clearly show that tissue- and class-specificity should be considered in future application of *GST* genes as biomarkers in biomonitoring.

## Conclusions

A *de novo* transcriptome of *P viridis* has been successfully generated; this transcriptome was based on the mRNA from three target tissues (adductor muscle, gills and hepatopancreas). Tissue-specific patterns were associated with transcriptome size, expression profiles of genes in GO terms, and subgroups of genes associated with stress responses and degradation of xenobiotics. Our findings provide an informative transcriptomic platform for further in-depth mechanistic environmental studies using the biomonitor *P. viridis*, and at the same time emphasizes the advantage of tissue-specific approaches in future investigations.

## Methods

### Sample collection and preparation

**Figure 4:**
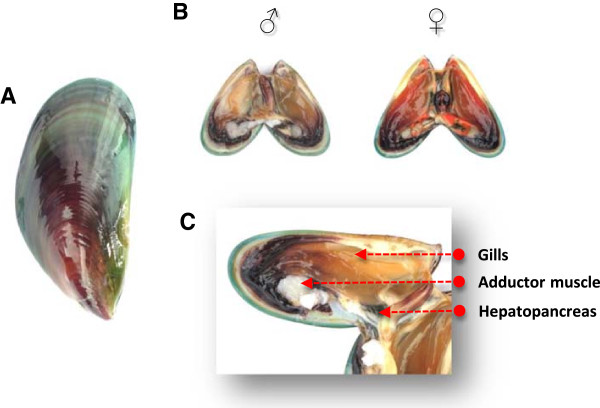
**Morphology of** ***Perna viridis*** **. (A)** The external morphology of the mussel. **(B)** The internal morphology of male and female mussels. **(C)** The three tissues examined were the gills, adductor muscle and hepatopancreas.

We obtained adult *P viridis* (4–5 cm shell length; Figure 4) for RNA sequencing from the field (field-type) and from mariculture farms. Animals from mariculture farms were exposed to various physical and chemical stressors (exposed-type) so as to obtain a wide spectrum of transcripts associated with environmental conditions. A similar approach was employed in the generation of other transcriptome databases [58]. According to the Animals (Control of Experiments) Ordinance, Chapter 340 (Department of Health, Hong Kong SAR), there is no requirement for permission to perform experiments involving mussels (invertebrate). The field-type samples were collected from different locations in Hong Kong waters. Locations encompassed the western (Butterfly Beach, 22°22'19.73"N and 113°57'38.31"E), southern (Po Toi, 22° 9'49.61"N and 114°15'7.73"E) and eastern waters (Tung Lung Island, 22°15'24.16"N and 114°17'13.76"E; Sam Mun Tsai, 22°27'8.87"N and 114°12'28.90"E) as each area had different hydrographic condition. The field-type samples were dissected to obtain target tissues (gills, adductor muscle and hepatopancreas) within 24 h of collection. Sex was determined by examining the external characteristic of the gonad, that is a female gonad was in bright orange brick red colour while male gonad was in milky colour [9].

The exposed-type mussels were obtained from a mariculture farm at Yung Shue O mariculture zone, Sai Kung, Hong Kong (22°25'26"N, 114°16'45"E). Acclimation and exposure treatments were conducted at the Swire Institute of Marine Science (SWIMS; The University of Hong Kong). Prior to exposure experiments, mussels were acclimated in running seawater for 7 days, and fed with the diatom *Thalassiosira pseudonana*. Mussels were then acutely exposed (24 h) to different individual model chemicals (metals, endocrine disruptors, organic pollutants and engineered nano-particles) and to different individual physical conditions (temperature, salinity, dissolved oxygen level and pH). Sublethal concentrations were used for chemical exposure, with concentrations set at the corresponding median effective concentration or 10% of the corresponding median lethal concentration available for *P. viridis* or other marine bivalves from the USEPA’s ECOTOX database (http://cfpub.epa.gov/ecotox/) and literatures. For the exposure experiments, mussels were treated with different types of chemical: metals, 730 μg/L CdCl_2_, (ECOTOX) and 65 μg/L CuSO_4_ (ECOTOX); endocrine disruptors, 0.32 μg/L triphenyltin chloride (TPTCl; ECOTOX) [59] and 40 μg/L dichlorodiphenyltrichloroethane (DDT; ECOTOX); organic pollutants, 5 μg/L polybrominated diphenyl ether 47 (PBDE 47; ECOTOX) [60] and 75 μg/L Benzo[a]pyrene (ECOTOX); and engineered nano particle, 13 mg/L nZnO (ECOTOX) designed to provide 600 μg/L zinc ions. For nZnO treatment, the concentration of zinc ions was estimated from the calculated dissolution rate of nZnO [61]. Mussels were kept in test solutions in 0.45-μm filtered artificial seawater (FASW; salinity: 30 ± 0.5‰; temperature: 25 ± 0.5°C and pH 8.0 ± 0.1). Organic compounds (TPTCl, DDT, PBDE 47 and Benzo[a]pyrene) were delivered into FASW using dimethylsulfoxide (DMSO) as a solvent at a final concentration of 0.01% (v/v).

Additionally, mussels were exposed to a range of: temperatures (15, 25 and 30°C) in FASW (30 ± 0.5‰; pH 8.0 ± 0.1); salinities (10, 20 and 30‰) in FASW (25 ± 0.5°C; pH 8.0 ± 0.1); dissolved oxygen (DO; 2.5 and 4.0 mg/L) in FASW (30 ± 0.5‰; 25 ± 0.5°C; pH 8.0 ± 0.1); and pH (7.5, 7.8 and 8.1) in FASW (30 ± 0.5‰; 25 ± 0.5°C). The mussels used in all treatments were kept at a density of one mussel per 250 mL of FASW. After 24 h of exposure, mussels were immediately dissected to obtain target tissues.

Two mussels, a male and a female, from each field site and from each exposure treatment (*n* = 44 mussels) were chosen for dissection to obtain the tissue samples of adductor muscle (ad), gills (g) and hepatopancreas (hp). These three types of tissue we examined were common targets that have been used in environmental studies [3]. Dissected tissue samples were immediately fixed in RNA*later*® (Applied Biosystems, Warrington, UK), and then stored at −20°C until required.

### RNA preparation and sequencing

Total RNA for each sample was extracted with the RNeasy® Mini Kit and digested with DNase I following the manufacturer’s instructions (QIAGEN, Hilden, Germany). Integrity and size distribution of RNA samples were verified using an Agilent 2100 Bioanalyser (Agilent Technologies, Germany). Samples with an RNA Integrity Number ≥ 8.0 were used for cDNA library preparation. The concentration of RNA in each extracted sample was measured using a Qubit 1.0 Fluorometer (Invitrogen, Carlsbad, USA).

Six cDNA libraries were generated from the three target tissues (male-hp, female-hp, male-g, female-g, male-ad, and female-ad), with each library comprising pooled samples of field-type and exposed-type mussels. The cDNA libraries were assigned to six individual sequencing lanes, and one dedicated PhiX control lane for matrix and phasing calculations was applied during sequencing. The cDNA libraries were prepared using Illumina**®** TruSeq RNA Sample Preparation Kits v2 (Catalog # RS-122-2001), and the Illumina sequencing was performed at the Centre of Genomic Sciences (The University of Hong Kong). In brief, poly-A containing mRNA was collected using poly-T oligo-attached magnetic beads. The purified mRNA was broken down into short fragments and was applied as a template to synthesize the first-strand cDNA using a random hexamer-primer and reverse transcriptase (SuperScript® II Reverse Transcriptase; Invitrogen, Catalog # 18064014). For second-strand cDNA synthesis, the mRNA template was removed and a replacement strand was generated to form double-stranded (ds) cDNA. The ds cDNA underwent end repair, 3’ adenylation and indexed adaptor ligation, and the adaptor-ligated libraries were enriched by 10 cycles of PCR. Qualified and quantified libraries were then sequenced using an Illumina Genome Analyzer IIx (GAIIx; Illumina, California, USA)*.* Image analysis and base calling was performed with SCS2.8/RTA1.8. FASTQ file generation and removal of failed reads were performed using CASAVA version 1.8.2.

### *De novo*assembly and annotation

The raw reads from the six samples were merged, and then each read was pre-processed by the trimming of adaptors and the removal of 10 bp from the 3’ end. Low-quality reads were filtered out based on three criteria: reads with more than 5% unknown “N” bases; reads having more than 50% bases with a quality value less than 10; and reads < 35 bp. The resulting high-quality reads were then used for *de novo* assembly with Trinity version trinityrnaseq_r2012-10-25 [62] using default parameters. Trinity used the greedy k-mer extension (k-mer 25) to assemble raw reads into unique sequences of transcripts, followed by clustering of the contigs to construct complete de Bruijn graphs, and finally processed the individual graphs to generate full-length transcripts. The assembled transcripts were further clustered using CD-HIT [63] to reduce redundancy. Briefly, CD-HIT employed a 'longest sequence first' algorithm by identifying a representative sequence and then clustered each subsequent sequence as a redundant or a new cluster sequence based on a similarity score set at ≥ 95% for our analysis. The longest transcript in each gene cluster defined by Trinity was selected as a “unigene” for downstream analysis.

The annotation of assembled transcripts/unigenes was performed based on sequence similarity searches using BLASTx against the NCBI nr database (08 February 2013), and BLASTn and tBLASTn searches against the EST databases from the molluscan genome projects of *Aplysia californica* (GenBank Accession Number AASC00000000, 255,605 EST entries), *Crassostrea gigas* (AFTI00000000, 206,647 EST entries) and *Lottia gigantea* (AMQO00000000, 252,091 EST entries), with an E-value threshold of 1 × 10^−5^ and a high-scoring segment pairs cut-off length of 33. Functional classification was carried out based on GO using Blast2GO [64]. GO terms were assigned and annotated to the transcripts/unigenes according to three main categories: cell component; molecular function; and biological process. The presence of a conserved domain in transcript/unigene was analysed using InterProScan [65] within Blast2GO. KEGG pathways were assigned to the transcripts using the online KEGG Automatic Annotation Sever (KAAS; http://www.genome.jp/kegg/kaas/) using the bi-directional best-hit method.

### Transcriptome quantification for individual tissues and sexes

The high-quality reads from the six libraries (2 sexes × 3 tissues) were individually mapped to the global assembled *de novo* trancriptome to estimate the abundance of transcripts/unigenes using RNA-Seq by Expectation Maximization (RSEM) version 1.2.3 [66] with a pipeline script provided by Trinity. High-quality reads were mapped to the transcripts and the resulting alignment file was inputted into RSEM to calculate the expression value for each transcript. Expression level was presented in a normalized expressed value as FPKM, which corrects for read differences in libraries and gene length. A transcript was only considered expressed when the FPKM cut-off value was ≥ 0.05.

### Real-time qPCR assays

Tissue-specific expression patterns for 15 selected genes (Additional file 10) associated with stress responses/detoxification were revealed using qPCR analysis. Mussel samples were chosen from selected treatments (i.e., control, 730 μg/L CdCl_2_, 13 mg/L nZnO, 40 μg/L DDT, and 0.32 μg/L TPTCl) of the exposed-type that used in the library generation. Primer pairs for qPCR assays were designed using Primer3 version 0.4.0 (http://bioinfo.ut.ee/primer3-0.4.0/). For amplification, 1 mM reverse and forward primers and 2 μL of cDNA were used per 20 μL reaction on a CFX96™ Real-Time System (Bio-Rad, Hercules, USA) with iQ™ *SYBR®* green supermix (Bio-Rad, Philadelphia, USA), following the manufacturer’s recommended protocol and default settings . Thermal cycling conditions involved 3 min of pre-heating at 95°C, followed by 40 cycles of amplification (10 s at 95°C, 30 s at 60°C); a melt curve analysis was performed after the 40th cycle (10 s at 95°C, then 65–95°C in 0.5°C increments every 5 s). Relative expression levels of target genes were calculated using the 2^-ΔΔC^_*T*_ method [67], with the *18S* rRNA gene used as a reference and pooled samples from all tissues as calibrator samples. The *18S* gene was shown to be a suitable reference gene in mussels and its expression was stable among different chemical treatments [18, 68]. Expression of the *18S* gene was not significantly different among the various control and treatment groups (ANOVA, *p* > 0.05), and between sexes (*t*-test, *p* > 0.05).

### Availability of supporting data

All supporting data are included as additional files. The raw sequencing reads were submitted to the NCBI Sequence Read Archive (Accession No. SRP043984).

## Electronic supplementary material

Additional file 1: **Summary of raw reads data for** ***Perna viridis.*** (PDF 328 KB)

Additional file 2: **BLAST top-hit species distribution for assembled transcripts of** ***Perna viridis.*** Note: *Hydra vulgaris* was formerly classified as *Hydra magnipapillata.* (PDF 321 KB)

Additional file 3: **Gene ontology (GO) annotations for the global transcriptome of** ***Perna viridis.*** GO terms were annotated at level 2 of classification according to three main categories (biological process, cellular component, and molecular function). (PDF 235 KB)

Additional file 4: **Gene ontology (GO) annotations for the transcriptome of individual tissues and by sex.** GO terms were annotated at level 2 of classification according to three main categories (biological process, cellular component, and molecular function). (PDF 1014 KB)

Additional file 5: **Distribution of genes from** ***Perna viridis*** **with putative protein annotations assigned to the KEGG pathways.** (PDF 240 KB)

Additional file 6: **Real-time qPCR analysis.** Relative mRNA expression levels of selected stress-associated genes from the gills, adductor muscle and hepatopancreas of *Perna viridis* after five different treatments (control; 730 μg/L CdCl_2_; 13 mg/L nZnO; 40 μg/L DDT; or 0.32 μg/L TPTCl). *HSP*, heat shock protein; *GSTa*, glutathione-S-transferase alpha-class; *GSTp*, glutathione-S-transferase pi-class; *GSTs*, glutathione-S-transferase sigma-class; *GSTo*, glutathione-S-transferase omega-class; and *CYP*, cytochrome p450. Expression levels are presented as mean relative mRNA expression level + S.E. (*n* = 4). Significant differences are denoted by uppercase letters (A, B and C) for tissues and lowercase letters (a, b and c) for treatments within each tissue (one-way ANOVA, SNK test; *p* < 0.05). (PDF 624 KB)

Additional file 7: **List of heat-shock protein (** ***HSP*** **) genes annotated in** ***Perna viridis.*** (XLSX 10 KB)

Additional file 8: **List of cytochrome p450 (** ***CYP*** **) genes annotated in** ***Perna viridis.*** (XLSX 14 KB)

Additional file 9: **List of glutathione S-transferase (** ***GST*** **) genes annotated in** ***Perna viridis.*** (XLSX 10 KB)

Additional file 10: **List of oligonucleotide primers used in real-time qPCR assays.** (PDF 272 KB)

## Abbreviations

Bp: Base pairs
Cd: Cadmium
CdCl_2_: Cadmium chloride
CD-HIT: Cluster Database at High Identity with Tolerance
C_T_: Threshold cycle
CuSO_4_: Copper (II) sulphate
*CYP*: Cytochrome P450
DDT: Dichlorodiphenyltrichloroethane
DMSO: Dimethylsulfoxide
DO: Dissolved oxygen
DOPA: 3, 4-dihydroxyphenyl-L-alanine
ds: Double-stranded
EST: Expressed Sequence Tag
FASW: Filtered artificial seawater
FPKM: Fragments Per Kilo bases per Million reads
GAIIx: Genome Analyzer IIx
GO: Gene Ontology
*GST*: Glutathione S-transferase
*HSP10*: 10 kDa heat shock protein
*HSP22*: Small heat shock protein 22
*HSP24.1*: Small heat shock protein 24.1
*HSP60*: Heat shock protein 60
*HSP70*: Heat shock cognate 70 kDa protein
*HSP71*: Heat shock 70 kDa protein 8
*HSP90*: Heat shock protein 90
*HSP*: Heat-shock protein
KASS: KEGG Automatic Annotation Sever
KEGG: Kyoto Encyclopedia of Genes and Genomes
N50: 50% of the length of the assembled sequences
NCBI nr database: National Center for Biotechnology Information Non-redundant database
NGS: Next-generation sequencing
nZnO: Nano zinc oxide
PBDE 47: Polybrominated diphenyl ether 47
PCO: Principle coordinate analysis plot
PCR: Polymerase chain reaction
Q30: Quality score of 30
RT-qPCR: Real-time quantitative PCR
RSEM: RNA-Seq by Expectation Maximization
SRA: High-throughput DNA and RNA sequence read archive
SWIMS: Swire Institute of Marine Science
TPT: Triphenyltin
TPTCl: Triphenyltin chloride
Zn: Zinc.
