# Supplementary material for: De novo transcriptome analysis of Perna viridis highlights tissue-specific patterns for environmental studies
Source: BMC Genomics. 2014 Sep 19;15(1):804. doi: 10.1186/1471-2164-15-804 (PMC4190305; doi:10.1186/1471-2164-15-804)
Supplement: Supplementary file 1 — Additional file 1: Summary of raw reads data for Perna viridis. (PDF 328 KB) [file 12864_2014_6498_MOESM1_ESM.pdf]

Additional file 1. Summary of raw reads data for *Perna viridis*.

**Item**	**Gills**		**Hepatopancreas**		**Adductor muscle**	
	**Male**	**Female**	**Male**	**Female**	**Male**	**Female**
**Throughput (Mb)**	8,545	9,889	10,320	8,563	9,097	10,352
**Number of reads**	84,607,612	97,908,328	102,182,018	84,784,834	90,074,178	102,494,014
**Sequence quality ≥ Q30**	87.60%	89.19%	85.09%	87.76%	85.39%	83.78%
**Mean quality score**	35	35	34	35	34	34
**GC %**	39%	39%	40%	40%	42%	42%
