# Supplementary material for: De novo transcriptome analysis of Perna viridis highlights tissue-specific patterns for environmental studies
Source: BMC Genomics. 2014 Sep 19;15(1):804. doi: 10.1186/1471-2164-15-804 (PMC4190305; doi:10.1186/1471-2164-15-804)
Supplement: Supplementary file 2 — Additional file 2: BLAST top-hit species distribution for assembled transcripts of Perna viridis. Note: Hydra vulgaris was formerly classified as Hydra magnipapillata. (PDF 321 KB) [file 12864_2014_6498_MOESM2_ESM.pdf]

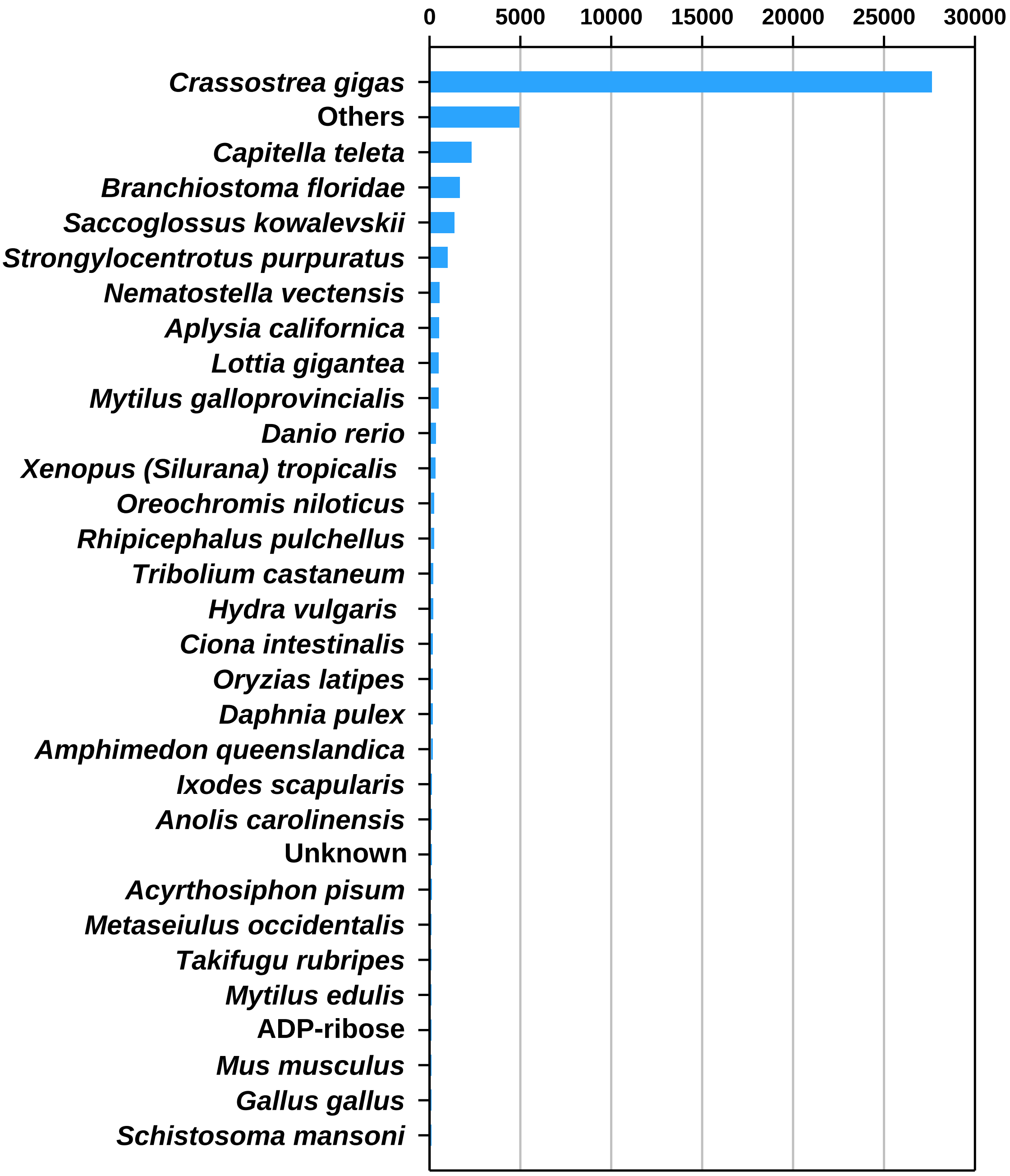

Additional file 2. BLAST top-hit species distribution for assembled transcripts of *Perna viridis*. Note: *Hydra vulgaris* was formerly classified as *Hydra magnipapillata.*
