# Supplementary material for: De novo transcriptome analysis of Perna viridis highlights tissue-specific patterns for environmental studies
Source: BMC Genomics. 2014 Sep 19;15(1):804. doi: 10.1186/1471-2164-15-804 (PMC4190305; doi:10.1186/1471-2164-15-804)
Supplement: Supplementary file 3 — Additional file 3: Gene ontology (GO) annotations for the global transcriptome of Perna viridis. GO terms were annotated at level 2 of classification according to three main categories (biological process, cellular component, and molecular function). (PDF 235 KB) [file 12864_2014_6498_MOESM3_ESM.pdf]

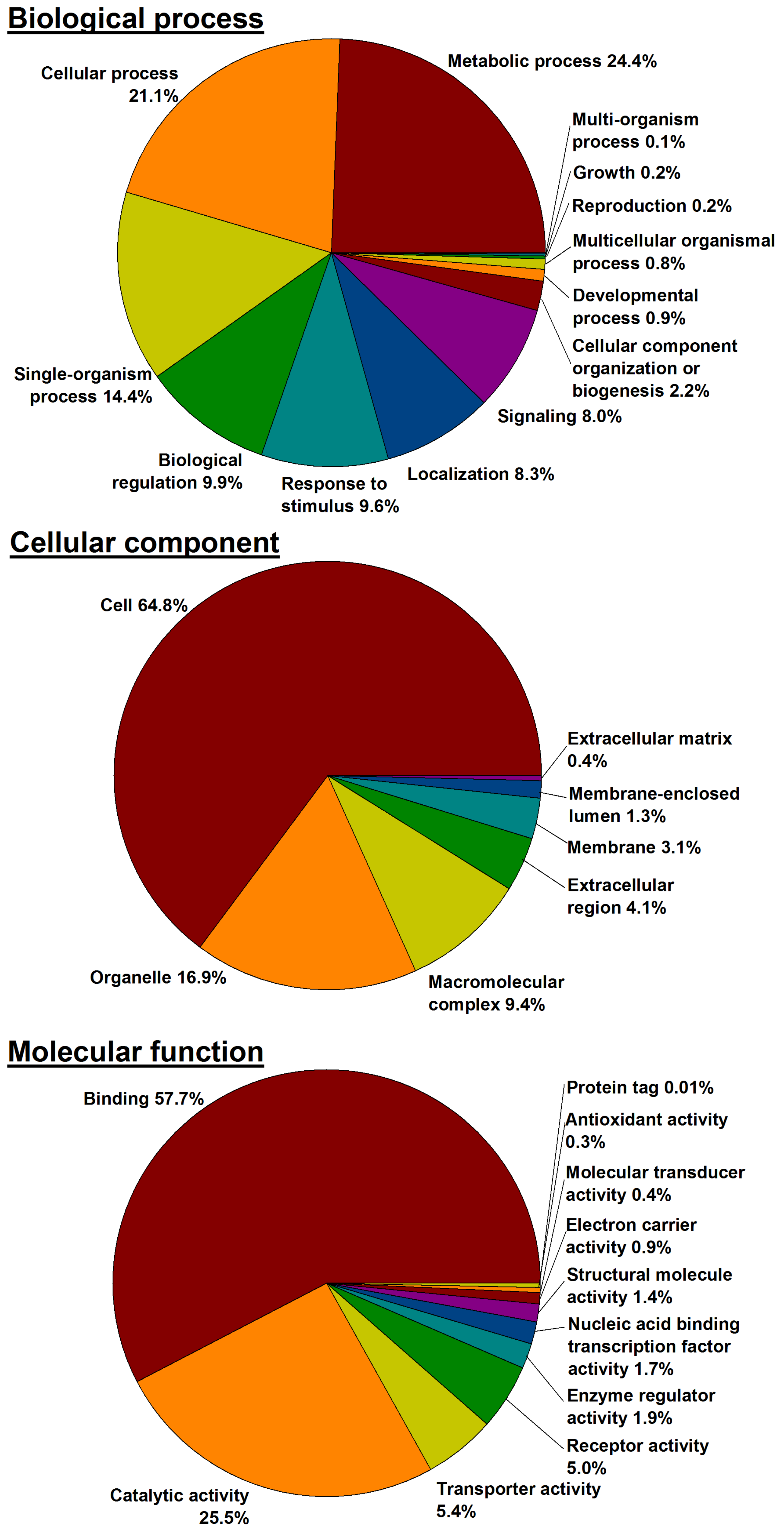

Additional_file_3: Gene ontology (GO) annotations for the global transcriptome of *Perna viridis*. GO terms were annotated at level 2 of classification according to three main categories (biological process, cellular component, and molecular function).
