# Supplementary material for: De novo transcriptome analysis of Perna viridis highlights tissue-specific patterns for environmental studies
Source: BMC Genomics. 2014 Sep 19;15(1):804. doi: 10.1186/1471-2164-15-804 (PMC4190305; doi:10.1186/1471-2164-15-804)
Supplement: Supplementary file 5 — Additional file 5: Distribution of genes from Perna viridis with putative protein annotations assigned to the KEGG pathways. (PDF 240 KB) [file 12864_2014_6498_MOESM5_ESM.pdf]

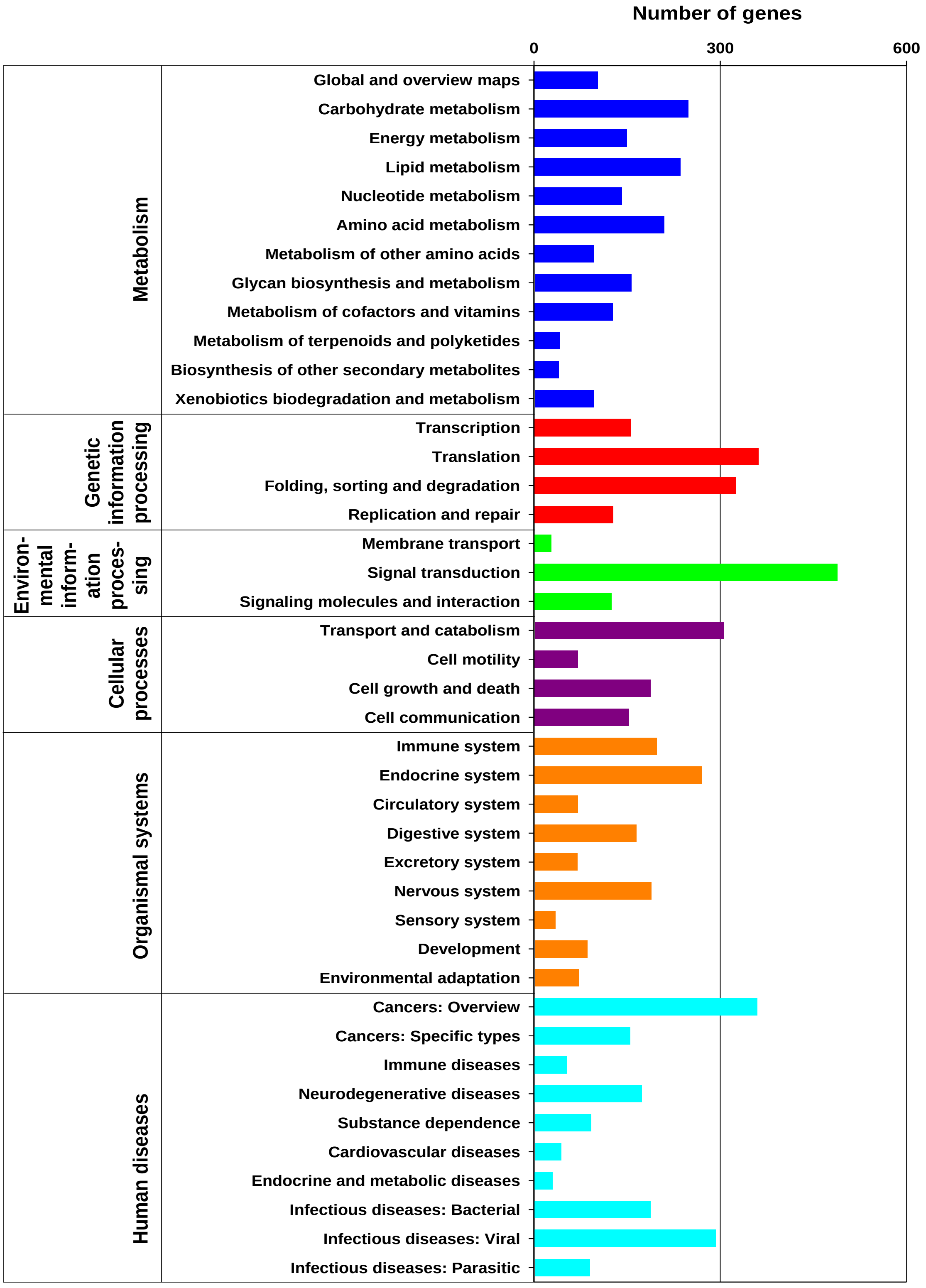

Additional file 5. Distribution of genes from *Perna viridis* with putative protein annotations assigned to the KEGG pathways.
