# Supplementary material for: De novo transcriptome analysis of Perna viridis highlights tissue-specific patterns for environmental studies
Source: BMC Genomics. 2014 Sep 19;15(1):804. doi: 10.1186/1471-2164-15-804 (PMC4190305; doi:10.1186/1471-2164-15-804)
Supplement: Supplementary file 6 — Additional file 6: Real-time qPCR analysis. Relative mRNA expression levels of selected stress-associated genes from the gills, adductor muscle and hepatopancreas of Perna viridis after five different treatments (control; 730 μg/L CdCl2; 13 mg/L nZnO; 40 μg/L DDT; or 0.32 μg/L TPTCl). HSP, heat shock protein; GSTa, glutathione-S-transferase alpha-class; GSTp, glutathione-S-transferase pi-class; GSTs, glutathione-S-transferase sigma-class; GSTo, glutathione-S-transferase omega-class; and CYP, cytochrome p450. Expression levels are presented as mean relative mRNA expression level + S.E. (n = 4). Significant differences are denoted by uppercase letters (A, B and C) for tissues and lowercase letters (a, b and c) for treatments within each tissue (one-way ANOVA, SNK test; p < 0.05). (PDF 624 KB) [file 12864_2014_6498_MOESM6_ESM.pdf]

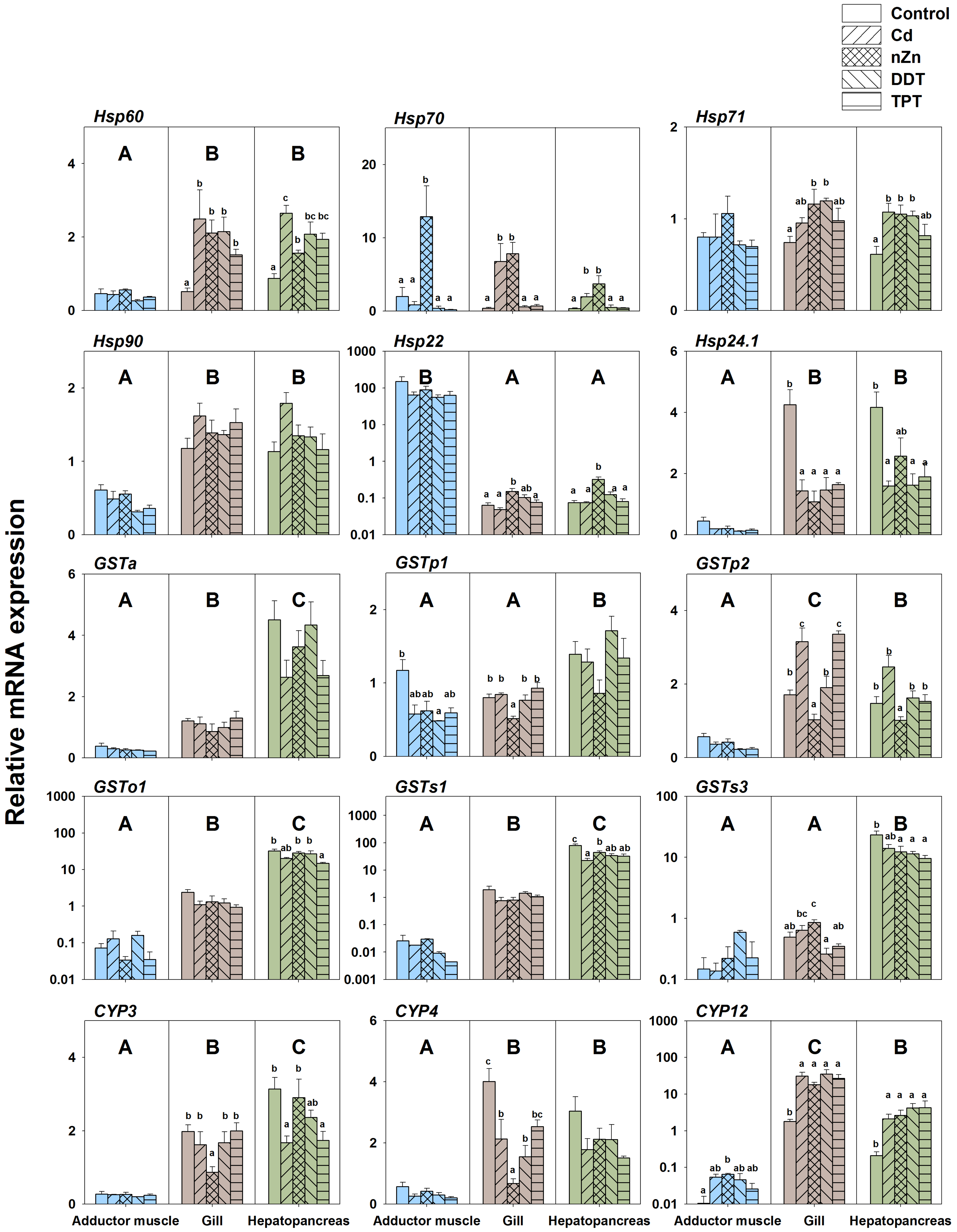

Additional_file_6: Real-time qPCR analysis. Relative mRNA expression levels of selected stress-associated genes from the gills, adductor muscle and hepatopancreas of *Perna viridis* after five different treatments (control; 730 μg/L $CdCl_2$; 13 mg/L nZnO; 40 μg/L DDT; or 0.32 μg/L TPTCl). *HSP*, heat shock protein; *GSTa*, glutathione-S-transferase alpha-class; *GSTp*, glutathione-S-transferase pi-class; *GSTs*, glutathione-S-transferase sigma-class; *GSTo*, glutathione-S-transferase omega-class; and *CYP*, cytochrome p450. Expression levels are presented as mean relative mRNA expression level + S.E. ($n = 4$). Significant differences are denoted by uppercase letters (A, B and C) for tissues and lowercase letters (a, b and c) for treatments within each tissue (one-way ANOVA, SNK test; $p < 0.05$).
