# Supplementary material for: De novo transcriptome analysis of Perna viridis highlights tissue-specific patterns for environmental studies
Source: BMC Genomics. 2014 Sep 19;15(1):804. doi: 10.1186/1471-2164-15-804 (PMC4190305; doi:10.1186/1471-2164-15-804)
Supplement: Supplementary file 10 — Additional file 10: List of oligonucleotide primers used in real-time qPCR assays. (PDF 272 KB) [file 12864_2014_6498_MOESM10_ESM.pdf]

Additional file 10. List of oligonucleotide primers used in real-time qPCR assays.

Gene ID	Gene name	Gene abbreviation		Primer sequence (5' to 3')
comp96782_c0_seq2	heat shock protein 60	*HSP60*	Forward:	GTACCAAAACATCGGGGCTAAA
			Reverse:	TGTGGCAGTTGTTGTTCCATCT
comp95233_c0_seq1	heat shock protein 70	*HSP70*	Forward:	ATCTGGTGATCGAAGTGATGCT
			Reverse:	AGTTTGAGATGCTTTGGTTGGT
comp76341_c0_seq1	heat shock protein 71	*HSP71*	Forward:	GACCAAGGAAACAGAACAACACC
			Reverse:	CTTTGCAGCGTCACCAATAAGA
comp76084_c0_seq1	heat shock protein 90	*HSP90*	Forward:	GGACAATTTGGTGTTGGCTTCT
			Reverse:	TGGTTCTCCTGTTGCAGGTTT
comp77735_c1_seq1	small heat shock protein 22	*HSP22*	Forward:	CCAACCTTTCGTGGAGGATTT
			Reverse:	CCTGGAGACTTTTCTTCGTGTTT
comp67005_c0_seq1	small heat shock protein 24.1	*HSP24.1*	Forward:	GGAAACTTCGTTAGCCGACA
			Reverse:	GACTTTGCCGTTCTCAACCTTC
comp73832_c0_seq1	Glutathione S-transferase alpha	*GSTa*	Forward:	GGCATGGGGTTTGTAGATGAA
			Reverse:	TCAGGAATTTGCAGGGTGACT
comp75555_c0_seq1	Glutathione S-transferase pi 1	*GSTp1*	Forward:	CCATACTGTCTCCTGGTTGTTTG
			Reverse:	CTGTTTGCCATTCCCATTGA
comp97391_c3_seq5	Glutathione S-transferase pi 2	*GSTp2*	Forward:	GAAAGCCCATACAGCCTCATACTT
			Reverse:	CTTGTCCGCCATTGTTGTGT
comp89293_c0_seq20	glutathione S-transferase sigma 1	*GSTs1*	Forward:	TTTAATGGGACGGGCAGAAC
			Reverse:	CTTGTCCAGCAGGTGTATTTGG
comp34627_c0_seq1	glutathione s-transferase sigma 3	*GSTs3*	Forward:	GAACTTGCTCGACTTCTCTTTGCT
			Reverse:	ATTTAACAGGTCCCCATTGCTC
comp64310_c0_seq1	Glutathione S-transferase omega 1	*GSTo1*	Forward:	TGTGCTTGGTTTTGTCCCTTC
			Reverse:	CTAATCCTCTCGGGTTCAGTTTC
comp81566_c0_seq4	cytochrome P450 family 3-like 3 protein	*CYP3*	Forward:	GTAGGAATGAGACTGGCTTTGG
			Reverse:	GTCACCGGAACCTCTGTTTTCT
comp90248_c0_seq1	cytochrome P450 family 4	*CYP4*	Forward:	ATCATCGGTAGACAGGCGTCA
			Reverse:	ACAGTTGGGTTGTGGTGTAAGG
comp56653_c0_seq1	cytochrome P450 12b2, mitochondrial	*CYP12*	Forward:	CAGCCAAGACATTCAACTCCA
			Reverse:	ACCTTTCATCCTTCATCGCAAC
Comp96598_c2_seq1	18S ribosomal RNA gene	*18S*	Forward:	TGGGAAAGAGCGCGTTTATTAG
			Reverse:	TGGGAAAGAGCGCGTTTATTAG
